# Does repeated exposure to hydrogen peroxide induce *Candida auris* resistance?

**DOI:** 10.1186/s13756-023-01281-5

**Published:** 2023-09-07

**Authors:** Luis Cobrado, Elisabete Ricardo, Patricia Ramalho, Angela Rita Fernandes, Acacio Goncalves Rodrigues

**Affiliations:** 1 Division of Microbiology, Department of Pathology, Faculty of Medicine, University of Porto, Al. Prof. Hernâni Monteiro, Porto, 4200 - 319 Portugal; 2 Center for Health Technology and Services Research / Rede de Investigação em Saúde, CINTESIS / RISE, University of Porto, Porto, Portugal; 3 Burn Unit, Department of Plastic and Reconstructive Surgery, University Hospital Center of São João, Porto, Portugal

**Keywords:** Hydrogen peroxide, Healthcare-associated infections, *Candida auris*, Biocides, Antimicrobial resistance.

## Abstract

**Background:**

To minimize environmental colonization by microorganisms that may persist and thrive in healthcare settings, thus reducing healthcare-associated infections (HAIs), new insights over already known biocides are certainly of relevance. Although the efficacy of hydrogen peroxide (H_2_O_2_) against the emergent yeast *Candida auris* is moderately documented, concerns over the potential induction of resistance after repeated exposure do persist. The main objective of the present study was to evaluate the hypothetical induction of *Candida auris* resistance following 30 days of consecutive exposure to lethal and sublethal concentrations of H_2_O_2_. Furthermore, the authors aimed to elucidate about the rank of efficacy of H_2_O_2_ against *C. auris* comparing to other *Candida* species and whether different strains of *C. auris* may display different susceptibilities to H_2_O_2_.

**Methods:**

During the induction of resistance assays, both type strains and clinical isolates of *Candida auris, Candida albicans* and *Candida parapsilosis* were exposed repeatedly to defined concentrations of H_2_O_2_, for 30 days.

**Results:**

After that period, no significant differences were found when comparing the minimal inhibitory concentration values of H_2_O_2_ in case of the induced strains *versus* each respective positive control. Moreover, H_2_O_2_ displayed similar effectiveness against all the tested *Candida* species and no differences were demonstrated among the distinct strains of *C. auris*.

**Conclusions:**

The adoption of H_2_O_2_ solutions in routine protocols in order to promote disinfection standards against *Candida auris*, improving patient safety and reducing healthcare costs, is certainly welcomed.

## Background

Presently, quite optimistic results regarding the prevention of healthcare-associated infections (HAIs) are being made available in the literature. However, the daily efforts of healthcare workers and further consistent scientific research are needed to fuel that trend. To minimize the colonization by infectious microorganisms able to persist and thrive in the healthcare environment, new strategies for cleaning and disinfection are being proposed, but with variable success [1–3]. Yet, no matter how appealing the new methods and biocide candidates may sound, new insights over already known and extensively used biocides are certainly welcomed and may play a relevant role towards the effective prevention of HAIs.

*Candida auris* is an emerging pathogen that has been isolated in several countries in a relatively short period of time, causing multiple outbreaks [4–11]. Even though both the aggregative and the non-aggregative phenotypic variants are able to form biofilm, the non-aggregative cell type has been more frequently isolated from infected patients, forming a more robust biofilm and displaying higher virulence [12–15]. *C. auris* has been associated with multidrug resistance, severe infections and high mortality rates [6, 8, 16]. Clinical isolates have been recovered most frequently from the blood of critically ill patients with candidemia, including cases associated with central venous catheter use, but also from patients with respiratory tract and urinary tract infections [5, 8, 9, 17]. Although several reports regarding nosocomial transmission have been published [8, 10, 16], data are still insufficient concerning the effectiveness of disinfectants against *C. auris* [18].

Currently, hydrogen peroxide (H_2_O_2_) is extensively used in healthcare settings, either as a liquid agent for surface disinfection, or as a vapour or aerosol for terminal disinfection and sterilization. The biocide action of H_2_O_2_ occurs through the generation of hydroxyl free radicals that damage essential cell components such as lipids, proteins and DNA, exhibiting a broad-spectrum activity against diverse bacteria, fungi and viruses [19–21]. However, the level of evidence of H_2_O_2_ efficacy against *C. auris* remains moderate [22] and, as far as it is known to the authors, no data exists addressing the potential induction of resistance after repeated use of H_2_O_2_.

Therefore, the main objective of the present study was to evaluate the potential induction of *C. auris* resistance following 30 days of consecutive exposure to lethal and sublethal concentrations of H_2_O_2_. Furthermore, it is to be clarified whether H_2_O_2_ is more effective against *C. auris* comparing to other *Candida* species and whether different strains of *C. auris* display different susceptibilities to H_2_O_2_.

## Methods

### Microbial strains and culture conditions

Details about the strains used in the study are displayed in Table 1. *Candida albicans* CA 016 and *Candida parapsilosis* CP 009 clinical strains were obtained from the strain collection of the Microbiology Division, Department of Pathology of the Faculty of Medicine, University of Porto, Portugal. Such strains were previously recovered from critical care patients. They were kept at -80ºC, in Brain Heart infusion broth with glycerol. Identification of clinical isolates was initially performed with Vitek system (bioMérieux, Vercieux, France) and confirmed by matrix-assisted laser desorption/ionization time-of-flight mass spectrometry (MALDI-TOF MS, Bruker).

*Candida albicans* ATCC 90,028 and *Candida parapsilosis* ATCC 22,019, from the American Type Culture Collection, were also used in the experiments. *C. auris* DSMZ 21,092 was obtained from the DSMZ-Deutsche Sammlung von Mikroorganismen und Zellkulturen GmbH. *C. auris* NCPF 8971 and *C. auris* NCPF 8977 were obtained from the Culture Collections Public Health England, National Collection of Pathogenic Fungi.

Following thawing, the yeast strains were initially maintained in Sabouraud Dextrose Agar (Liofilchem, Italy), at 37 °C. For growth in liquid media, YPD (Yeast Peptone Dextrose broth medium, Liofilchem, Italy) was used; strains were cultivated at 37 °C, 120 rpm, constant temperature in an incubator shaker.

**Table 1:** Details about the strains used in the study

Species	Strains	Notes
*Candida albicans*	ATCC 90,028	Reference/Type strain
	CA 016	Clinical strain resistant to fluconazole
*Candida parapsilosis*	ATCC 22,019	Reference/Type strain
	CP 009	Clinical strain resistant to caspofungin
*Candida auris*	DSMZ 21,092	Reference strain; first isolate of *C. auris*
	NCPF 8971	Reference strain; does not aggregate in suspension
	NCPF 8977	Reference strain; forms aggregates in suspension

### H_2_O_2_ susceptibility testing

Initial (day 0) minimal inhibitory concentrations (MICs) of H_2_O_2_ (Labkem, Barcelona, Spain) were determined for all strains (Table 1), adapted from protocols M27-A3 and supplement M27-S4 by the Clinical Laboratory for Standards Institute (CLSI) [23–25]. Briefly, a stock solution of H_2_O_2_ (30%) was used to prepare serial two-fold dilutions of H_2_O_2_ solutions in RPMI 1640 (Sigma – Aldrich, St Louis, USA) and the final concentrations tested ranged from 0.875 to 0.00171%. Inocula were prepared from 24 h cultures, according to the same protocols, i.e., a stock suspension was initially prepared with a concentration of 1 × 10^6^ to 5 × 10^6^ cells per mL, corresponding to a 0.5 McFarland standard. Then, a working suspension was prepared by a 1:100 dilution followed by a 1:20 dilution of the stock suspension with RPMI 1640 broth medium, which resulted in 5.0 × 10^2^ to 2.5 × 10^3^ cells per mL. MICs were determined in 96-well plates, adding to each well 100 µl of the final inoculum and 100 µl of the respective serial dilution of the H_2_O_2_ solution. Positive control wells containing only inoculum and RPMI 1640 medium and negative control wells containing the lowest concentration of H_2_O_2_ solution and RPMI 1640 medium were included. For each strain, 2 replicates were performed for the determination of MIC values of hydroxide peroxide, and this determination was performed twice in different days. Results were read after 24 and 48 h of incubation at 35ºC. MIC values were defined as the lowest concentration of H_2_O_2_ which inhibited microbial growth > 99%.

### In vitro induction of resistance to H_2_O_2_

After MIC determination, *Candida* strains were repeatedly exposed to different concentrations of H_2_O_2_. Briefly, a single, randomly selected colony from a 24 h fresh culture (Sabouraud agar medium) was suspended in 10 mL of YPD (Yeast Peptone Dextrose broth medium) and incubated at 35 °C, 150 rpm, overnight. Afterwards, 1mL of each culture was resuspended into 9 mL of fresh YPD medium, in the presence of different concentrations of H_2_O_2_ according to MIC values previously determined; a positive control for each strain was included, i.e., 1 mL of each culture in fresh medium with no H_2_O_2_; a negative control containing only YPD medium was also included and manipulated to check for contamination. Concentrations tested corresponded to 2xMIC, MIC, 1/2xMIC for all strains (Table 2). Every 24 h, 1 mL of each culture was resuspended into 9 ml of fresh medium containing the same concentration of H_2_O_2_ (along with the respective positive control), for a 30-day period. From each daily sub-culture, an aliquot was stored at -80 °C in BHI with 40% glycerol. Every 2 days, a 10 µl loopful of yeast cells was cultured in Sabouraud agar plates to check for culture contamination. Every 10 days along the 30 day duration of the assay, H_2_O_2_ MIC was determined according to CLSI protocols [23–25]. During the induction period, strain identification was confirmed at days 10, 20 and 30 of incubation by MALDI –TOF.

## Results

### Induction of resistance to H_2_O_2_ assessment

The MICs of H_2_O_2_ corresponding to the induced strains and their controls are detailed in Table 2. After 30 days of incubation with H_2_O_2_, MIC values ranged between 0.007% and 0.055% for all strains. In the vast majority, no difference was found between MICs of H_2_O_2_ solution in the case of the induced strain and its respective positive control. In a minority of cases (*C. parapsilosis* CP 009 and *C. auris* NCPF 8971), at day 30, a non-significant difference of 1 single dilution was found between MIC of H_2_O_2_ solution in the case of the induced strain *versus* its respective positive control.

**Table 2:** MICs of H_2_O_2_ (%) corresponding to the induced strains of yeasts and respective controls

Species	Strains	H_2_O_2_	Day 0	Day 10	Day 20	Day 30
* C. albicans*	ATCC 90,028	Control	0,014	0,014	0,014	0,014
		1/2MIC		0,014	0,014	0,014
		MIC		0,014	0,014	0,014
		2MIC		0,014	0,014	0,014
	CA 016	Control	0,014	0,014	0,028	0,014
		1/2MIC		0,014	0,028	0,014
		MIC		0,014	0,028	0,014
		2MIC		0,014	0,028	0,014
* C. parapsilosis*	ATCC 22,019	Control	0,014	0,014	0,028	0,014
		1/2MIC		0,014	0,028	0,014
		MIC		0,014	0,028	0,014
		2MIC		0,014	0,028	0,014
	CP 009	Control	0,007	0,014	0,028	0,014
		1/2MIC		0,014	0,028	0,014
		MIC		0,014	0,028	0,014
		2MIC		0,014	0,028	0,014
* C. auris*	NCPF 8971	Control	0,014	0,028	0,055	0,028
		1/2MIC		0,028	0,055	0,028
		MIC		0,028	0,055	0,028
		2MIC		0,028	0,055	0,028
	NCPF 8977	Control	0,014	0,028	0,028	0,014
		1/2MIC		0,028	0,028	0,014
		MIC		0,028	0,028	0,014
		2MIC		0,028	0,028	0,014
	DSMZ 21,092	Control	0,014	0,014	0,028	0,014
		1/2MIC		0,014	0,028	0,014
		MIC		0,014	0,028	0,014
		2MIC		0,014	0,028	0,014

## Discussion

The search for more efficient protocols to clean and disinfect surfaces of healthcare settings is constant and certainly driven by novel methods and technology, with much relevant published work on the subject [1, 2, 26–31]. However, much more scarcely do novel substances with disinfectant claims arise in the market. Therefore, new insights over extensively used disinfectants are mostly welcomed.

The test method chosen to study the exposure of pathogenic yeasts to H_2_O_2_ for a long period of time has obvious limitations, such as the underestimation of the real MICs since H_2_O_2_ is an oxidizing agent stable in solution, but unstable in the presence of organic molecules such as those present in the media used for determining the MICs. Besides, differences in the MIC values were smaller than expected because no dramatic genetic shifts were expected to occur by the action of H_2_O_2_. Taken all this together, the most relevant points of the study will be highlighted.

Firstly, even though H_2_O_2_ has demonstrated to be microbicidal against several bacteria, viruses, yeasts and spores, its activity against different strains of *C. auris* and concerns about the potential induction of microbial resistance after repeated exposure do subsist. Since yeasts may persist in the environment for weeks to months [32], most certainly due to ineffective cleaning and disinfection protocols and to the emergence of resistance to biocides, the current study addressed the potential of *Candida* organisms to develop resistance following prolonged exposure. The authors tested *Candida* spp frequently associated to HAIs, many of them with a high propensity to colonize hospital settings and persist in the environment for a long time, as is the case with *C. parapsilosis* and *C. auris*. MIC values ranged between 0.007% and 0.055%, which are considerably lower than the concentrations of solutions of H_2_O_2_ used in disinfection in real healthcare settings. Moreover, during the 30 days of the experiment, non-significant differences were found between MICs of the induced strains and their respective positive controls.

Secondly, regardless *C. auris* had been previously described as displaying different resistance to H_2_O_2_ in comparison to *C. albicans* [33–35], such was not the case in this study. In fact, a similar resistance pattern was found for the three yeast species, as demonstrated by the non-significant differences in MIC values.

Finally, in contrast with a previous publication [36], the effectiveness of H_2_O_2_ against *C. auris* did not differ significantly depending on the *C. auris* strains (Table 2).

## Conclusions

H_2_O_2_ did not induce yeast resistance during the test period, in particular regarding the emergent and alarming *C. auris* (either aggregative or non-aggregative strains), which is undoubtedly good news for a biocide with such an extensive use in healthcare scenarios. The adoption of H_2_O_2_ solutions in routine protocols to improve disinfection standards, promoting patient safety and reducing healthcare costs, is certainly highly welcomed.

## Data Availability

All the data supporting conclusions are available in Tables 1 and 2.

## References

[1] Assadian O, Harbarth S, Vos M, Knobloch JK, Asensio A, Widmer AF (2021). Practical recommendations for routine cleaning and disinfection procedures in healthcare institutions: a narrative review. J Hosp Infect.

[2] Dancer SJ, King MF (2021). Systematic review on use, cost and clinical efficacy of automated decontamination devices. Antimicrob Resist Infect Control.

[3] Dancer SJ, Kramer A (2019). Four steps to clean hospitals: LOOK, PLAN, CLEAN and DRY. J Hosp Infect.

[4] Satoh K, Makimura K, Hasumi Y, Nishiyama Y, Uchida K, Yamaguchi H (2009). Candida auris sp. nov., a novel ascomycetous yeast isolated from the external ear canal of an inpatient in a japanese hospital. Microbiol Immunol.

[5] Lee WG, Shin JH, Uh Y, Kang MG, Kim SH, Park KH (2011). First three reported cases of nosocomial fungemia caused by Candida auris. J Clin Microbiol.

[6] Chowdhary A, Sharma C, Duggal S, Agarwal K, Prakash A, Singh PK (2013). New clonal strain of Candida auris, Delhi, India. Emerg Infect Dis.

[7] Magobo RE, Corcoran C, Seetharam S, Govender NP (2014). Candida auris-associated candidemia, South Africa. Emerg Infect Dis.

[8] Calvo B, Melo AS, Perozo-Mena A, Hernandez M, Francisco EC, Hagen F (2016). First report of Candida auris in America: clinical and microbiological aspects of 18 episodes of candidemia. J Infect.

[9] Schelenz S, Hagen F, Rhodes JL, Abdolrasouli A, Chowdhary A, Hall A (2016). First hospital outbreak of the globally emerging Candida auris in a european hospital. Antimicrob Resist Infect Control.

[10] Vallabhaneni S, Kallen A, Tsay S, Chow N, Welsh R, Kerins J (2016). Investigation of the First Seven reported cases of Candida auris, a globally emerging invasive, multidrug-resistant fungus - United States, May 2013-August 2016. MMWR Morb Mortal Wkly Rep.

[11] Ruiz Gaitan AC, Moret A, Lopez Hontangas JL, Molina JM, Aleixandre Lopez AI, Cabezas AH (2017). Nosocomial fungemia by Candida auris: first four reported cases in continental Europe. Rev Iberoam Micol.

[12] Sherry L, Ramage G, Kean R, Borman A, Johnson EM, Richardson MD (2017). Biofilm-Forming capability of highly virulent, multidrug-resistant Candida auris. Emerg Infect Dis.

[13] Kean R, Delaney C, Sherry L, Borman A, Johnson EM, Richardson MD et al. Transcriptome Assembly and Profiling of Candida auris reveals novel insights into biofilm-mediated resistance. mSphere. 2018;3(4). 10.1128/mSphere.00334-18 PMC6041501 29997121

[14] Singh R, Kaur M, Chakrabarti A, Shankarnarayan SA, Rudramurthy SM (2019). Biofilm formation by Candida auris isolated from colonising sites and candidemia cases. Mycoses.

[15] Du H, Bing J, Hu T, Ennis CL, Nobile CJ, Huang G (2020). Candida auris: Epidemiology, biology, antifungal resistance, and virulence. PLoS Pathog.

[16] Arauz AB, Caceres DH, Santiago E, Armstrong P, Arosemena S, Ramos C (2018). Isolation of Candida auris from 9 patients in Central America: importance of accurate diagnosis and susceptibility testing. Mycoses.

[17] Jeffery-Smith A, Taori SK, Schelenz S, Jeffery K, Johnson EM, Borman A et al. Candida auris: a review of the literature. Clin Microbiol Rev. 2018;31(1). 10.1128/CMR.00029-17 PMC5740969 29142078

[18] Ku TSN, Walraven CJ, Lee SA (2018). Candida auris: disinfectants and implications for infection control. Front Microbiol.

[19] Ikai H, Odashima Y, Kanno T, Nakamura K, Shirato M, Sasaki K (2013). In vitro evaluation of the risk of inducing bacterial resistance to disinfection treatment with photolysis of hydrogen peroxide. PLoS ONE.

[20] Kampf G. Antibiotic ResistanceCan be enhanced in Gram-Positive species by some Biocidal Agents used for disinfection. Antibiot (Basel). 2019;8(1). 10.3390/antibiotics8010013 PMC6466599 30717270

[21] Kampf G. Biocidal Agents used for Disinfection can enhance Antibiotic Resistance in Gram-Negative species. Antibiot (Basel). 2018;7(4). 10.3390/antibiotics7040110 PMC6316403 30558235

[22] Cadnum JL, Shaikh AA, Piedrahita CT, Sankar T, Jencson AL, Larkin EL (2017). Effectiveness of Disinfectants against Candida auris and other Candida Species. Infect Control Hosp Epidemiol.

[23] Clinical and Laboratory Standard Institute. Reference method for broth dilution Antifungal susceptibility testing of yeasts. Approved standard, M27-A3. 2008. CLSI, Wayne, PA, USA.

[24] Clinical and Laboratory Standard Institute (2012). Reference method for broth dilution Antifungal susceptibility testing of yeasts; fourth informational supplement. Approved standard, M27-S4.

[25] Clinical and Laboratory Standards Institute (2009). Methods for dilution antimicrobial susceptibility tests for bacteria that grow aerobically - eighth edition: approved standard M07-A8.

[26] Boyce JM (2021). A review of wipes used to disinfect hard surfaces in health care facilities. Am J Infect Control.

[27] Deshpande A, Donskey CJ (2017). Practical approaches for Assessment of Daily and Post-discharge Room Disinfection in Healthcare Facilities. Curr Infect Dis Rep.

[28] Cobrado L, Silva-Dias A, Azevedo MM, Rodrigues AG (2017). High-touch surfaces: microbial neighbours at hand. Eur J Clin Microbiol Infect Dis.

[29] Weber DJ, Kanamori H, Rutala WA (2016). No touch’ technologies for environmental decontamination: focus on ultraviolet devices and hydrogen peroxide systems. Curr Opin Infect Dis.

[30] Boyce JM (2016). Modern technologies for improving cleaning and disinfection of environmental surfaces in hospitals. Antimicrob Resist Infect Control.

[31] Weber DJ, Anderson D, Rutala WA (2013). The role of the surface environment in healthcare-associated infections. Curr Opin Infect Dis.

[32] Kramer A, Schwebke I, Kampf G (2006). How long do nosocomial pathogens persist on inanimate surfaces? A systematic review. BMC Infect Dis.

[33] Pathirana RU, Friedman J, Norris HL, Salvatori O, McCall AD, Kay J et al. Fluconazole-Resistant Candida auris is susceptible to salivary histatin 5 killing and to intrinsic host defenses. Antimicrob Agents Chemother. 2018;62(2). 10.1128/AAC.01872-17 PMC5786754 29158282

[34] Day AM, McNiff MM, da Silva Dantas A, Gow NAR, Quinn J. Hog1 regulates stress tolerance and virulence in the Emerging Fungal Pathogen Candida auris. mSphere. 2018;3(5). 10.1128/mSphere.00506-18 PMC6200985 30355673

[35] Zatorska B, Moser D, Diab-Elschahawi M, Ebner J, Lusignani LS, Presterl E (2021). The effectiveness of surface disinfectants and a micellic H(2)O(2) based water disinfectant on Candida auris. J Mycol Med.

[36] Abdolrasouli A, Armstrong-James D, Ryan L, Schelenz S (2017). In vitro efficacy of disinfectants utilised for skin decolonisation and environmental decontamination during a hospital outbreak with Candida auris. Mycoses.

